# Thermodynamics of Isomers and Solubility Prediction in Multicomponent Sugar Solutions

**DOI:** 10.1021/acs.jpcb.4c08616

**Published:** 2025-03-31

**Authors:** Silvio Trespi, Shina Roshanfekr, Marco Mazzotti

**Affiliations:** Institute of Energy and Process Engineering, ETH Zurich, 8092 Zurich, Switzerland

## Abstract

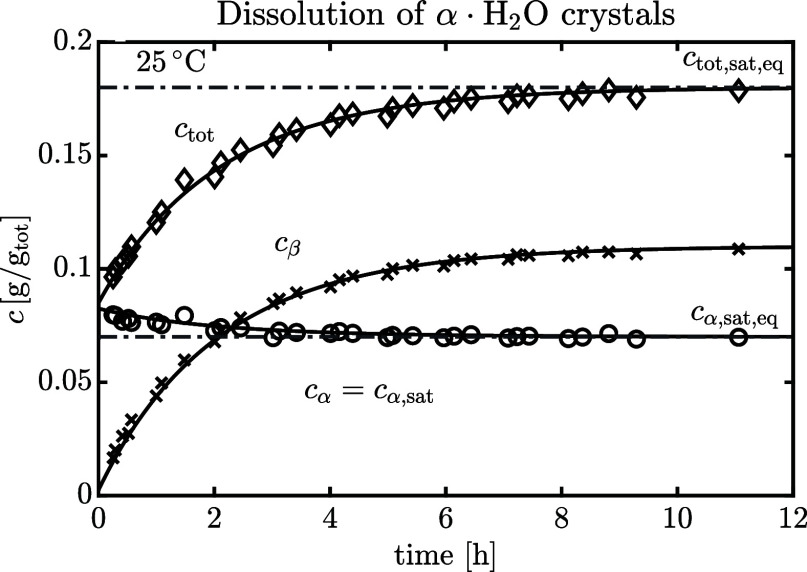

A rigorous thermodynamic modeling framework for a system of isomers in chemical equilibrium is developed and applied to the lactose-water system. Through the knowledge of the water activity and of the liquid phase composition, thermodynamically consistent expressions for the activity coefficients of lactose isomers have been derived and used in the context of solid–liquid equilibria to predict the dependence of the saturation concentration of α-lactose on the dissolved β-lactose concentration. We also developed a comprehensive first-principles model that accurately describes the dissolution dynamics of α-lactose monohydrate. The data support the hypothesis that the sugar activity coefficients are a stronger function of the total sugar content rather than of the sugar solution composition. The activity coefficient expressions have been used to quantitatively predict the effect of glucose, galactose, and sucrose on the solubility of α-lactose monohydrate.

## 1 Introduction

The field of “isomer group thermodynamics”, a theoretical framework that involves treating isomers as pseudospecies by grouping them together, was developed by Smith^1^ and expanded by Alberty.^2^ This approach was initially applied to complex gaseous hydrocarbon mixtures and, more recently, to the field of biothermodynamics, where the solutions are typically dilute and where the primary source of nonideality consists of electrostatic interactions. In concentrated solutions, however, solute–solute interactions or preferential solvation phenomena play an important role. This is the case of multicomponent aqueous sugar solutions, where multiple isomers coexist in chemical equilibrium. There are plenty of examples coming from the food industry: fruit juices are mainly constituted of sucrose, glucose, and fructose.^3^ Aqueous mixtures of sucrose and lactose are the raw material for the production of powdered ice cream.^4^ Hydrolysis of lactose yielding equimolar mixtures of glucose and galactose is an established industrial process to enhance the sweetness of the mixture.^5,6^ The apparently binary solutions glucose-water or lactose-water are actually ternary solutions where two sugar isomeric forms, α and β, are in chemical equilibrium through a reaction known as mutarotation.^7,8^ In the fructose-water system, there are three isomers present in nonnegligible amounts.^9^

Sugar crystallization, one of the most important unit operations in the food industry, yields preferentially one specific isomer of the mixture depending on the operating conditions.^29^ Hence, rigorous phase equilibria calculations must distinguish between isomers in the liquid phase. Visser^10^ investigated the dependence of the saturation concentration of α-lactose on the dissolved β-lactose. However, its direct determination is not straightforward because β-lactose isomerizes in solution to α-lactose. Therefore, he took advantage of the experimental evidence reported by Solstad^11^ on sucrose solubility that “all sugars depress sucrose solubility in the same way” and measured the decrease in saturation concentration of α-lactose brought about by dissolving progressively larger amounts of sucrose in the aqueous mixture. Although the equality in solubility depression caused by β-lactose and sucrose has never been proven, Hodges et al.^12^ used it to model the dissolution dynamics of α-lactose monohydrate, whereas other authors^13,14^ used it to define the driving force for α-lactose monohydrate crystallization.

Building on the isomer group thermodynamics concept, this contribution aims to fill some of these research gaps by the application of fundamental thermodynamic equations. The manuscript is organized as follows: Section 2 describes the experimental setup used in this study; Section 3 describes a novel thermodynamic model for a system of isomers in chemical equilibrium and applies it to water-lactose solutions; the parameters are calibrated using water activity and liquid phase composition measurements. The theory is applied in Section 4 to model the dissolution of α-lactose monohydrate in water and to uncover the effect on solubility due to dissolved β-lactose. Finally, in Section 5, the thermodynamic model is demonstrated to be able to predict the influence of additional dissolved sugars other than β-lactose (galactose, glucose, sucrose) on α-lactose monohydrate solubility.

## 2 Materials and Methods

### 2.1 Chemicals

Ultrapure deionized water (Milli-Q AdvantageA10 system, Millipore, Zug, Switzerland) was used. α-Lactose monohydrate (CAS Number 5989-81-1, BioXtra, ≥ 99% total lactose (GC), ≤4% β-lactose) was purchased from Sigma-Aldrich.

### 2.2 Experimental Setup and Procedures

Preparation of lactose solutions and dissolution experiments were conducted in a 100 mL automated, temperature-controlled glass reactor (EasyMax 102, Mettler Toledo, Greifenbach, Switzerland). Rubber stoppers were used to seal the reactor lid openings, thereby creating a closed environment where the water vapor content remained independent of the laboratory’s relative humidity. Water activity measurements were performed using an AQUALAB TDL2 water activity meter (METER Group Inc., Pullman, WA) on 5–10 mL samples of filtered solution, with a filter pore size of 0.22 μm. Prior to each set of measurements, the device calibration was tested using two standard solutions with water activities lower and higher than those of the samples to be analyzed: depending on the concentration of the lactose solution, the water activity standards used were 1.000, 0.984, and 0.92. All measurements were conducted at 25 °C. The quantification of dissolved α- and β-lactose was performed with liquid chromatography following an experimental protocol described recently.^8^ Concentrated lactose samples were filtered using a 0.22 μm filter and diluted with a known mass of deionized water prior to injection.

## 3 Isomer Thermodynamics

### 3.1 Theory

Consider a system of *N* isomers dissolved in a generic solvent such as water. If chemical equilibrium is established, the chemical potential of each isomer is equal, i.e.,
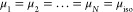
1where μ_iso_ is defined as the chemical potential of the isomer group.

Taking into account the (*N* – 1) chemical reaction constraints (*NR* = *N* – 1) in the single-phase system (*P* = 1), the Gibbs phase rule states that the number of degrees of freedom of the system, *F*, for a system consisting of *NC* = *N* + 1 components equals
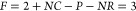
2Hence, the intensive state of the system is fully determined once three independent intensive variables have been specified, in this case, *T*, *P,* and *x*_w_.

The Gibbs–Duhem relationship imposes a constraint on the variation of the intensive variables:
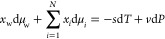
3

At constant temperature and pressure, eqs 1 and 3 imply that
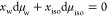
4where  is the molar fraction of the isomer group. Therefore, by treating all of the isomers as a single pseudospecies, the system can be analyzed as if it were a binary system, i.e., consisting of the isomer group and the solvent, e.g., water. At constant temperature,
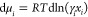
5

Considering eqs 4 and 5, which can be used to estimate the thermodynamically consistent activity coefficient of the isomer group from measurements of the activity coefficient of water by integration, yields
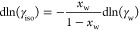
6

The application mentioned above is commonly described in thermodynamics textbooks^15,16^ as a method for determining the activity coefficient of a nonvolatile solute dissolved in a solvent, such as sucrose in a binary sucrose–water mixture. The integration of eq 6 can be performed analytically when the 2-suffix Margules expression is used for the activity coefficient of water (defined using the mole fraction as the concentration scale and using the pure liquid at the temperature of the system and at an arbitrary pressure as the standard state):
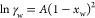
7

Here, *A* is the empirical constant of the mixture, which depends on temperature but is independent of composition. This is an empirical model that describes a parabolic dependence of the excess Gibbs free energy on the composition of a binary mixture. It has been shown to provide a good representation for many simple (e.g., argon/oxygen, benzene/cyclohexane) and complex (e.g., sugar/water, acetic acid/water) mixtures.^3,17^ Integration of eq 6 using eq 7 yields the following formula for the activity coefficient of the isomer group (defined using as standard state an ideal dilute solution extrapolated to pure solute, hence the asterisk as superscript *)^17^:
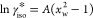
8

When dealing with isomers in chemical equilibrium, an additional term that takes into account the isomer composition arises. Focusing on isomer *i*, eq 4 can be written as
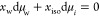
9

Using eqs 5 and 7 and introducing the equilibrium molar fraction ratio *K*_x_ to express each isomer molar fraction as a function of *x*_*i*_ as *x*_*j*_ = *K*_x,*ji*_*x*_*i*_, the final expression for dln γ_*i*_ is reported below (details of the derivation are in Supporting Information, Section 1):
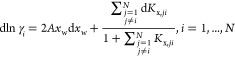
10

If *N* > 2, the integration of the second term of eq 10 is not straightforward as it relies on the relationship between each *K*_x,*ji*_ and *x*_w_ (the case *N* = 3 is discussed in the Supporting Information, Section 1). If *N* = 2, the integral is analytical and independent of the specific functional relationship that links *K*_x_ and *x*_w_.

### 3.2 Analytical Solution for Two Isomers

Eq 10 for the special case of two isomers (*K*_x_ = *x*_2_/*x*_1_) can be written as
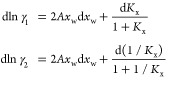
11

Since the isomers can be considered solutes dissolved in water, it is advantageous to express the activity coefficients using as the standard state the ideal dilute solution, extrapolated to pure solute (also known as the solute standard state). Eq 11 can be analytically integrated for each isomer *i* using as the initial state the infinite dilution in water, where ():
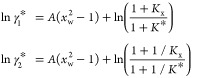
12

K* is the thermodynamic equilibrium constant for isomerization 1 *⇌* 2, where the solute standard state is used for both isomers. Subtracting one from the other:
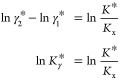
13confirming that the expressions reported by eq 12 are thermodynamically consistent with each other. Since the activity coefficients approach unity in the limit of infinite dilution (and, consequently, also their ratio ), the thermodynamic equilibrium constant K* can be estimated^18,19^ by plotting *K*_x_ for different amounts of solute dissolved and then extrapolating to pure water (*x*_w_ → 1).

The activity coefficient of the isomer group is expressed using eq 8 and corresponds to a corrected geometric average of the single isomer activity coefficients:
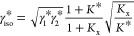
14

### 3.3 Water-Lactose Solutions in Mutarotation Equilibrium

In this contribution, the water-lactose system has been experimentally investigated: it is a ternary mixture, where two isomers, α- and β-lactose, interconvert one into the other according to a reaction known as mutarotation. Defining α as isomer 1 and β as isomer 2, eq 12 describes how the activity coefficients depend on *x*_w_, where *K*_x_(*x*_w_) = *x*_β_/*x*_α_ is the equilibrium molar fraction ratio. The estimation of  and  (where the subscript underlines that the activity coefficients are estimated under mutarotation equilibrium) through eq 12 requires:

- the parameter *A* of the 2-suffix Margules expression from water activity measurements; and
- the thermodynamic equilibrium constant of mutarotation, K*, and the functional relationship between the equilibrium molar fraction ratio and the dissolved lactose concentration or, equivalently, the molar fraction of water, i.e., *K*_x_ = *K*_x_(*x*_w_).

**Figure 1:**
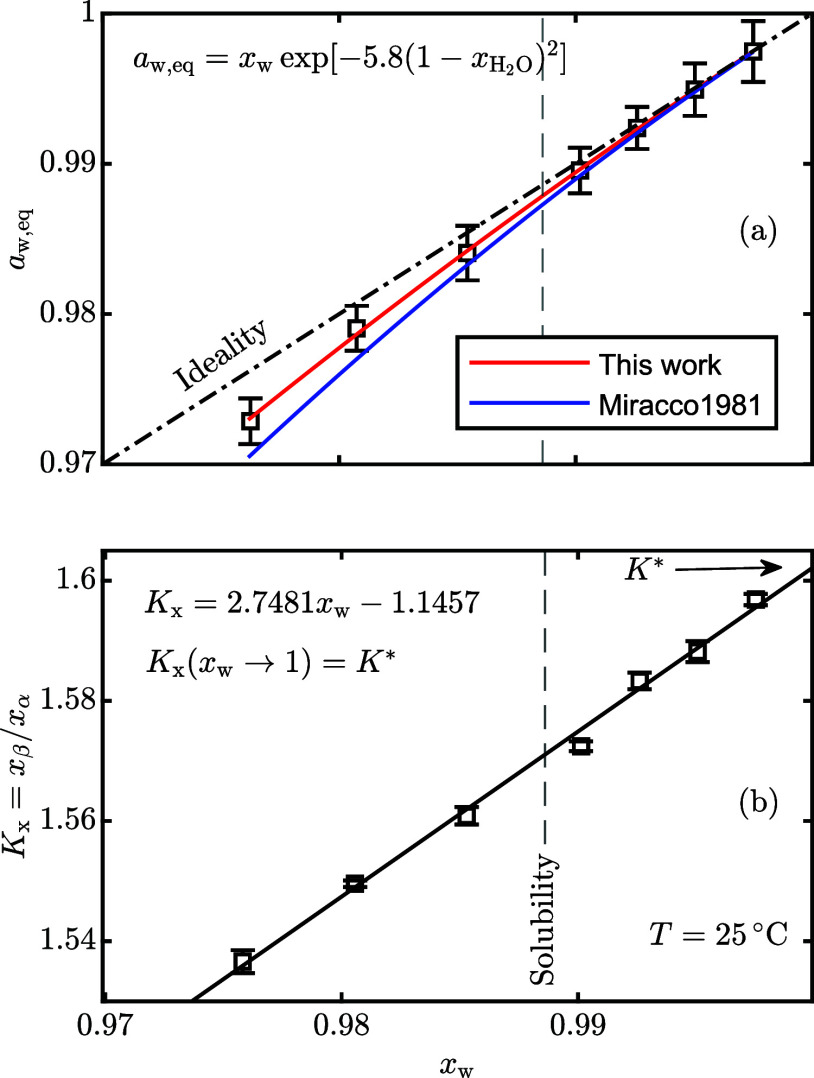
(a) Water activity and (b) equilibrium molar fraction ratio of aqueous lactose solutions as a function of the molar fraction of water at 25 °C. The vertical dashed line corresponds to the solubility of α-lactose monohydrate. The error bars refer to ±1 standard deviation.

Figure 1 shows the water activity (a) and the equilibrium molar fraction ratio (b) of lactose solutions up to  at 25 °C. Given that the solubility at 25 °C is , corresponding to a water molar fraction of 0.9886, measurements at higher concentrations have been accomplished by heating the suspension to completely dissolve the powder and by subsequently cooling back to 25 °C. Due to its slow nucleation rate, lactose supersaturated solutions remain metastable long enough (8–12 h) to reach mutarotation equilibrium at 25 °C.

As shown in Figure 1a, the water activity measurements are accurately described by a 2-suffix Margules expression (eq 7) using *A* = −5.8 ± 0.5 (where the uncertainty is the standard error from linear regression):
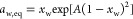
15

Supersaturated solutions exhibit significant negative deviations from ideal solution behavior, indicative of stronger water–lactose interactions than the lactose–lactose or water–water ones. Miracco et al.^20^ reported a smaller value, namely, *A* = −10.3, but they measured lactose solutions up to solubility only at 25 °C. It is interesting to observe from Figure 1b that on increasing the lactose concentration (i.e., decreasing the water molar fraction), the equilibrium molar fraction ratio, *K*_x_, slightly decreases. The trend is consistent with the experimental results reported in our earlier paper:^8^*K*_x_ decreases when decreasing the water molar fraction by the addition of a cosolvent in diluted lactose solutions. The thermodynamic equilibrium constant K* is obtained from *K*_x_(*x*_w_) by linear extrapolation to an infinite dilution (*x*_w_ → 1).

**Figure 2:**
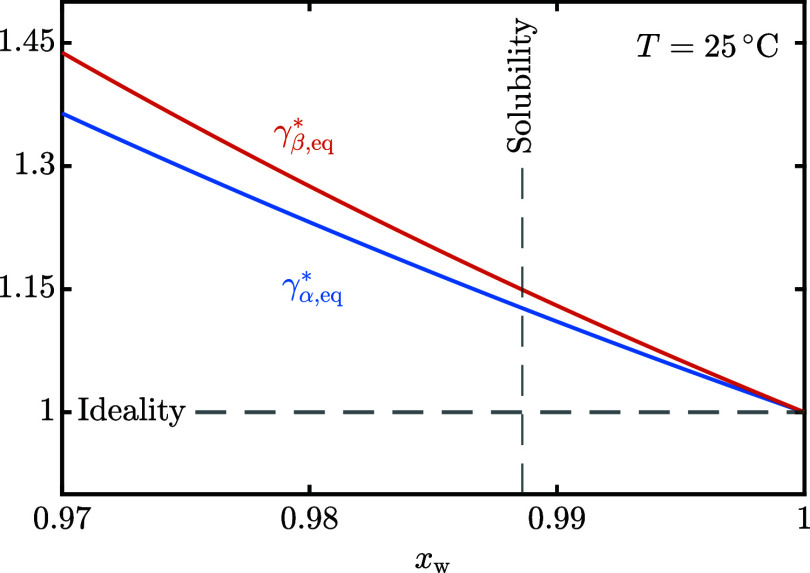
Activity coefficient of α- and β-lactose at mutarotation equilibrium as a function of the water molar fraction, *x*_w_.

Eq 12 estimates the activity coefficients of α- and β-lactose, reported as a function of *x*_w_ in Figure 2. Increasing the lactose concentration enhances the solute–solute interactions and suppresses the solute–solvent interactions at the same time, thus increasing the activity coefficients. In particular,  grows faster than , thus leading to the slightly decreasing trend of *K*_x_ in Figure 1b. This is representative of the slightly different solvation behaviors of the lactose isomers. It is worth underlining that, if *K*_x_ did not change with the lactose concentration, *K*_γ_^*^ would be equal to 1 and therefore . At the highest lactose concentration tested (),  is 1.05 instead.

## 4 Water-Lactose Solutions Not in Mutarotation Equilibrium

The activity coefficient expressions of eq 12 depend only on the molar fraction of water because they have been derived under the assumption of a solution in mutarotation equilibrium.

To test their validity in solutions not in mutarotation equilibrium, we experimentally investigated and modeled the isothermal dissolution dynamics of α-lactose monohydrate. The reaction scheme is as follows:
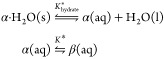
16

**Figure 3:**
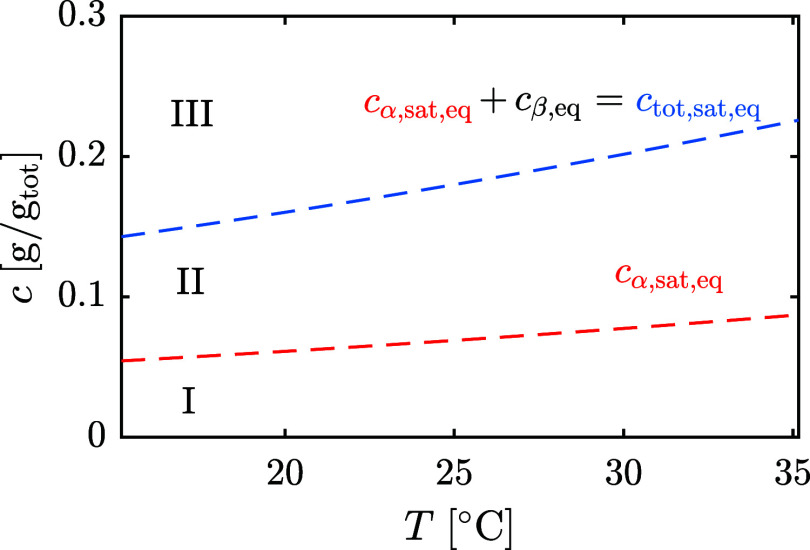
Solubility diagram of α-lactose monohydrate in water, showing the dissolved α- and total lactose concentrations at saturation and in mutarotation equilibrium as a function of temperature.

When the α-lactose monohydrate powder comes in contact with water, a two-step process begins: the fast dissolution of the powder enriches the system in α-lactose only, followed by the slow mutarotation reaction converting the α-lactose into β-lactose until mutarotation equilibrium is reached. At fixed temperatures, the phase diagram reported in Figure 3 defines three different system responses^12^ depending on the ratio between the initial powder loading and the solvent mass (i.e., the initial suspension density).

If the initial suspension density is smaller than *c*_α,sat,eq_, the behavior of the system will be described as Type I. The powder dissolves within seconds, yielding a clear solution, and the system slowly evolves toward mutarotation equilibrium. These conditions have been used earlier^8^ to estimate the kinetics of lactose mutarotation. If, instead, the initial suspension density is larger than *c*_α,sat,eq_, then the fast dissolution guarantees the establishment of solid–liquid equilibrium within seconds. Two cases have to be distinguished. If the initial suspension density is smaller than *c*_tot,sat,eq_, the system will eventually become undersaturated due to mutarotation, and the whole powder will dissolve (Type II). If the initial suspension density is larger than *c*_tot,sat,eq_, the system remains a saturated suspension, while mutarotation equilibrium is reached (Type III).

### 4.1 On the Dissolution of α-Lactose Monohydrate Crystals

#### 4.1.1 Modeling

Accurately modeling the dissolution of α-lactose monohydrate requires assumptions regarding the size and shape of the crystals. The crystal population is generally nonuniform and characterized by a number-weighted size distribution, *n*(*L*), defined per unit mass of suspension. Each crystal is assumed to have a characteristic length, *L*, and a volumetric shape factor, *k*_v_, such that the crystal volume is given by *k*_v_*L*^3^. For a system where the crystal shape remains consistent across the population, a single *k*_v_ value can be used for all crystals. In the case of approximately spherical crystals, where *L* represents the sphere’s diameter, the volumetric shape factor is given by *k*_v_ = π/6.

The total mass of suspended crystals, *m*_cry_, is directly related to the third moment of the size distribution function, *n*(*L*), as follows:
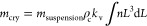
17where ρ_c_ is the crystal density. The dissolution of α-lactose monohydrate crystals brings about a stoichiometrically equal release of water and α-lactose as described by eq 16: for each gram of α-lactose monohydrate crystals dissolved, χ = *M*_α_/*M*_α·H_2_O_ = 0.95 g of α-lactose is released. The model used to describe the dissolution of α-lactose monohydrate powder entails a mass balance equation for each of the solution components (α-lactose, β-lactose, and water) and a population balance equation for the crystals:
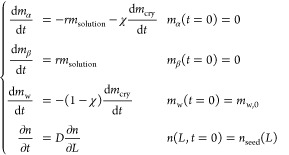
18

Mutarotation in the liquid phase is described by a first-order reversible reaction, *r*, referenced to the mass of solution *m*_solution_, where the forward kinetic constant has been estimated in diluted conditions^8^ and thermodynamic consistency is ensured through the equilibrium molar fraction ratio, *K*_x_:
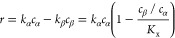
19where *c*_α_ and *c*_β_ are the weight fractions in solution. The population balance equation for the crystals assumes that the dissolution rate, *D*, is not a function of the crystal size, *L*, and that there is negligible agglomeration and breakage. Following the work by Bötschi et al.,^21^ the dissolution rate is modeled using a linear driving force:
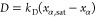
20

The dissolution rate is always positive because (*x*_α,sat_ – *x*_α_) > 0; hence, the initial seed population, *n*_seed_, shifts toward smaller particle sizes. The solubility product of α-lactose monohydrate is reported below as the product of the water and α-lactose activities at saturation (assuming the activity of the α-lactose monohydrate solid to be unity):
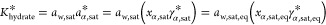
21

At a given temperature, the solubility product is constant.^22^ In particular, its value can be computed from standard solubility measurements with the excess solute method, letting the suspension reach simultaneously solid–liquid equilibrium and mutarotation equilibrium. While *x*_α,sat,eq_ can be directly measured by analysis of the liquid phase,  and *a*_w,sat,eq_ can be estimated using eqs 12 and 15, yielding . Away from mutarotation equilibrium,  is used in eq 21 to estimate *x*_α,sat_ and to define the driving force for dissolution in eq 20 under the assumption (to be verified a posteriori) that
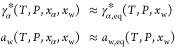
22meaning that  and *a*_w_ are a stronger function of the total dissolved lactose concentration (i.e., of the water molar fraction) rather than of the specific isomeric ratio between α- and β-lactose. This is a reasonable hypothesis, as water is the dominant component on a molar basis and water–sugar mixtures exhibit negative deviations from ideality, indicative of heterotypic (water–sugar) interactions that are stronger than homotypic interactions (water–water or sugar–sugar). Hence, β–β, β–α, or α–α interactions play a minor role with respect to sugar hydration in the energetics of the system. Therefore,
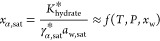
23The system dynamics is controlled by the ratio of the dissolution rate to the mutarotation rate. In the specific case of α-lactose monohydrate, dissolution is significantly faster than mutarotation,^12^ and hence the solid–liquid equilibrium is established significantly faster than mutarotation equilibrium. Therefore, an accurate description of lactose dissolution does not require an estimation of the dissolution kinetic constant, *k*_D_, because, neglecting a very short initial transient in the order of seconds (discussed more in detail in the Supporting Information, Section 2), the system is always at solid–liquid equilibrium. In comparison, the establishment of mutarotation equilibrium is a slow process and requires 12 h at 25 °C. Hence, detailed knowledge of the kinetics, namely, *k*_α_ and *k*_β_, is mandatory. These have been estimated in one of our previous works,^7^ whereas *k*_D_ has been chosen after a sensitivity analysis on the model output to guarantee a significantly faster dissolution rate compared to mutarotation. The model is solved numerically with a code developed in-house, using the method of characteristics to treat the population balance partial differential equation. Time integration is performed with the MATLAB ode113 solver. It employs the Adams–Bashforth–Moulton predictor-corrector method for numerical integration. This variable-step, variable-order algorithm adapts dynamically to the rate of change of the dependent variable, adjusting the integration order between 1 and 13 to find an optimal compromise between accuracy and computational time.^23^ The parameter values for the simulations are listed in Table 1.

**Table 1:** Parameter Values Used for Simulating the Dissolution of α-Lactose Monohydrate at 25 °C

parameter	symbol	value [unit]
crystal density	ρ_c_	1545 [kg m^–3^]
volumetric shape factor	*k*_v_	π/6 [−]
mass fraction of α-lactose in α ·H_2_O crystals	χ	0.95 [−]
forward mutarotation kinetic constant	*k*_α_	0.64 [h^–1^]
dissolution kinetic constant	*k*_D_	10^–3^ [ms^–1^]
solubility product of α ·H_2_O		0.00494 [−]

The crystal density is from Visser.^10^ The forward mutarotation kinetic constant is from our previous work.^8^ The seed crystal particle size distribution, *n*_seed_, is chosen to be Gaussian with a mean of 10 μm and a standard deviation of 1 μm.

#### 4.1.2 Comparison with Experiments and Discussion

**Figure 4:**
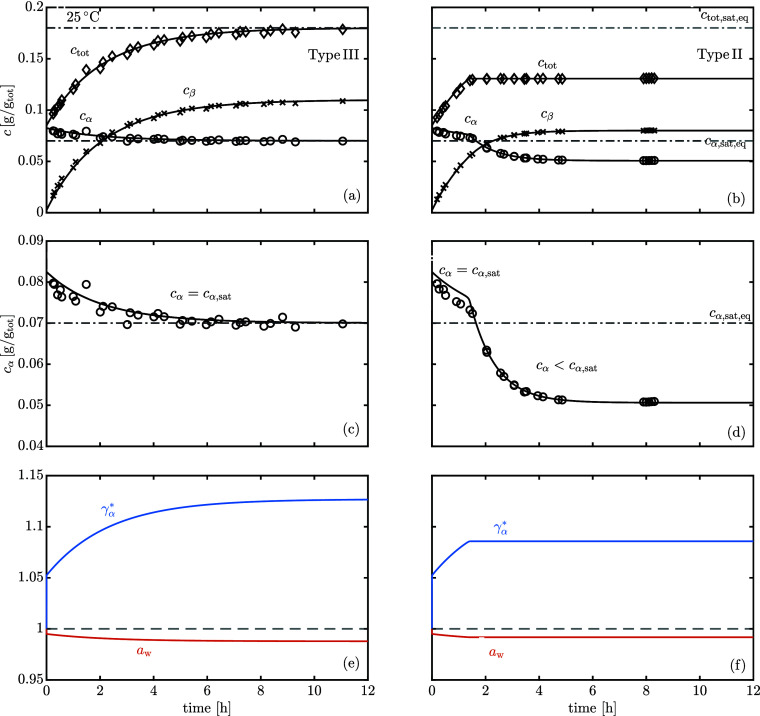
Experimental and modeling results of α-lactose monohydrate dissolution in water at 25 °C. Symbols represent experimental data, and solid lines refer to model prediction. Pictures (a), (c), and (e) refer to the Type III experiments where excess powder with respect to *c*_tot,sat,eq_ is added. Pictures (b), (d), and (f) refer to the Type II experiments where excess powder with respect to *c*_α,sat,eq_ is added, but not enough to reach *c*_tot,sat,eq_.

The model output and experimental data for Type III and II experiments at 25 °C are reported in Figure 4, using an initial suspension density of 0.23 and . In particular, Figure 4a–d compares the model predictions and the experimental data in terms of α-lactose, β-lactose, and total lactose concentrations. Figure 4e,f reports the evolution of the water activity and of the α-lactose activity coefficient. It is important to emphasize that Figure 4 shows the long-term mutarotation-controlled dissolution of α-lactose monohydrate, where experimental data are available. The short initial transient is discussed in detail in 2. It is clear that the model accurately describes the dissolution dynamics of α-lactose monohydrate without any additional fitting parameter. In particular:

- In Type III experiments (Figure 4a), *c*_tot_ approaches the total lactose solubility at mutarotation equilibrium at 25 °C, i.e., . A lower value corresponding to the initial suspension density is approached in Type II experiments (Figure 4b).
- The mutarotation kinetics estimated in diluted conditions^8^ accurately describes the dynamics of *c*_β_ toward mutarotation equilibrium (Figure 4a,b).
- In both Type III and Type II experiments, *c*_α_ approaches its solubility value, *c*_α,sat_ (Figure 4c,d), after a very short initial transient (discussed in detail in the Supporting Information Section 2); the first experimental measurements (10 min after the powder comes in contact with water) confirm that *c*_α_ is already at its saturation value, indicative of the significantly faster dissolution compared to mutarotation. The decreasing trend in *c*_α,sat_ over time can be explained by considering eq 21 and by looking at Figure 4e,f, where the increase in  is dominant over the reduction of *a*_w_. It is important to underline that estimating  using eq 12 is strictly valid only under mutarotation equilibrium and not during the whole dissolution process. However, the good agreement between the model and the data supports the hypothesis used to write eq 22. In Type II experiments (Figure 4d), the solid completely dissolves after indicatively 1.4 h and *c*_α_ departs from *c*_α,sat_, approaching mutarotation equilibrium.

### 4.2 On the Solubility Dependence of α-Lactose Monohydrate on β-Lactose

The aforementioned thermodynamics-based model quantitatively describes lactose dissolution without any fitting parameter. However, in the literature, the decrease of *x*_α,sat_ as mutarotation proceeds (Figure 4c) has been discussed by Visser,^10^ who proposed the empirical parameter *F* > 0 to model it (on a molar basis):
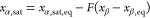
24

However, *x*_β_ could not be varied independently of *x*_α_ because of the chemical equilibrium between the lactose isomers, making the estimation of *F* not straightforward. Based on the experimental evidence^11,24^ that “sugars depress each other's solubilities in the same way”, Visser^10^ measured *F* from the decrease of *x*_α,sat_ caused by the addition of glucose or sucrose to lactose solutions as if they were β-lactose, assuming that the effect of adding a foreign sugar on the equilibrium ratio of the lactose isomers is negligible. If *F* is expressed on a mass per mass of water basis, it is the same for sucrose and glucose systems and supports the original hypothesis to use it in the lactose-water system through eq 24. However, it is important to point out that *F* is not the same for sucrose and glucose systems when expressed on a molar fraction or weight fraction basis (Supporting Information Section 3). This occurs because, although *F* is dimensionless, it relates the concentration of two solutions with different compositions and, therefore, different molecular weights.

The rigorous thermodynamic framework introduced in Section 3.2 can be used to provide an expression to estimate the empirical parameter *F*. Since *x*_α,sat_ ≈ *f*(*x*_w_) (eq 23), expanding in the Taylor series around *x*_w_ = *x*_w,eq_ and retaining only the first term yield
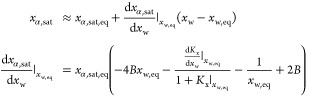
25By comparing eq 25 with eq 24 and recalling that *x*_w_ – *x*_w,eq_ = −(*x*_β_ – *x*_β,eq_) – (*x*_α,sat_ – *x*_α,sat,eq_), the thermodynamic framework estimates *F* (molar fraction basis) as follows:
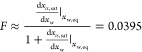
26that compares favorably with the values estimated by Visser^10^ from the solubility depression of α-lactose monohydrate caused by the addition of sucrose, equal to 0.0434. The influence of foreign sugars on lactose solubility is further investigated in the following section.

## 5 Influence of Foreign Sugars on Lactose Solubility

Eq 22 is based on the hypothesis that *a*_w_ and  do not depend on the specific isomeric ratio between β- and α-lactose but only on the total sugar content, and it is supported by comparison with experimental dissolution data. Building on this concept, a broader-impact hypothesis on aqueous sugar systems is that any additional sugar component, though not in chemical equilibrium with α-lactose, has a negligible direct influence on both *a*_w_ and , as long as the reduction in the water molar fraction is taken into account. This indirect contribution increases  and decreases *a*_w_. If the contribution of  is dominant, as already shown for the simple lactose-water system in Figure 4e,f, it would at least qualitatively explain the empirical observation^10,11,24^ that “sugars depress each other's solubilities in the same way”.

**Figure 5:**
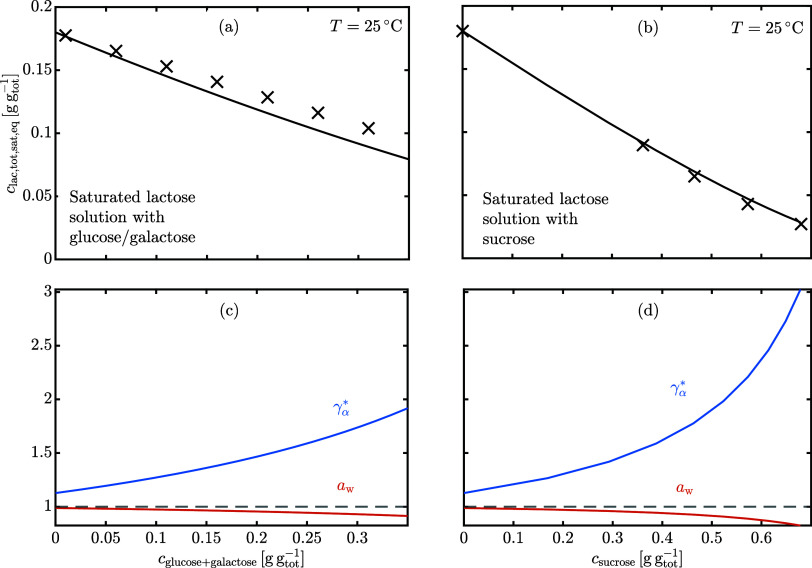
Measurements^24,25^ and model predictions of total lactose solubility depression caused by the addition of (a) glucose/galactose or (b) sucrose at 25 °C. The corresponding α-lactose activity coefficient and water activity are reported in (c) and (d).

Figure 5 shows the experimental^24,25^ total lactose solubility depression caused by either glucose/galactose (a) or sucrose (b) dissolved. The solid lines are the model predictions, where eq 23 and *K*_x_(*x*_w_) (Figure 1b) are used with the same parameters calibrated for water-lactose solutions to estimate  and *a*_w_, reported in Figure 5c,d. The reason behind the quantitative agreement with sucrose experiments and the only qualitative agreement with glucose/galactose experiments is most probably the Margules constant, *A*. Baeza et al.^26^ recently reported *A*_sucrose_ = −6.01 and *A*_glucose_ = −1.77 at 25 °C. This is consistent with the experimental evidence that the Margules constant for binary sugar-water solutions increases (in absolute value) with the mean number of equatorial hydroxyl groups.^27,28^ Compared to our estimation for lactose (*A*_lactose_ = −5.8), we confirm the experimental trend in terms of the Margules constant and argue that all disaccharides, like sucrose, will produce the same solubility depression on α-lactose monohydrate that is naturally caused by β-lactose because they have a similar Margules constant. Monosaccharides or oligosaccharides will still decrease lactose solubility, but the prediction using lactose-water parameters is less accurate.

## 6 Conclusions

Thermodynamically consistent activity coefficient expressions for two isomers dissolved in a solvent have been derived under the assumption of chemical equilibrium and shown to be a function of the solvent molar fraction only at fixed temperature and pressure. A comprehensive and thermodynamically consistent dissolution-mutarotation model has been built and applied to the lactose-water system. The model predictions and experimental data are in perfect agreement, supporting the conclusions (i) that mutarotation is considerably slower compared to the establishment of solid–liquid equilibrium by dissolution and (ii) that the activity coefficient of α-lactose is a stronger function of the total sugar content of the solution rather than of the specific sugar composition. The presence of foreign sugars other than α-lactose, such as β-lactose, glucose, galactose, or sucrose, increases , driving the α-lactose saturation concentration down, allowing quantitative thermodynamic-based predictions of sugar solubility in multicomponent sugar solutions using only pseudobinary lactose-water data.

## Supporting Information Available

Additional details on the derivation of the activity coefficient expression; dissolution modeling: approaching solid–liquid equilibrium; estimation of *F* according to Visser; and experimental data on the effect of foreign sugars on lactose solubility (PDF)
