# Supplementary material for: Thermodynamics of Isomers and Solubility Prediction in Multicomponent Sugar Solutions
Source: J Phys Chem B. 2025 Mar 31;129(14):3661–9. doi: 10.1021/acs.jpcb.4c08616 (PMC11995376; doi:10.1021/acs.jpcb.4c08616)
Supplement: Supplementary file 1 — jp4c08616_si_001.pdf [file jp4c08616_si_001.pdf]

Supporting Information

Thermodynamics of isomers and Solubility Prediction in Multicomponent Sugar Solutions

Silvio Trespi, Shina Roshanfekr, Marco Mazzotti

Institute of Energy and Process Engineering, ETH Zurich, 8092 Zurich, Switzerland

1 Additional details on the derivation of the activity coefficient expression

The stoichiometry constraint for the liquid phase mole fractions is:

$$x_{\mathrm{w}} + \sum_{j=1}^{N} x_j = x_{\mathrm{w}} + x_{\mathrm{iso}} = 1 \tag{S1}$$

The Gibbs-Duhem equation is reported in Equation (9). The differential of the chemical potential at constant temperature is reported in Equation (5). The activity coefficient of water is experimentally well described by the 2-suffix Margules expression, namely Equation (7). plugging Equation (5) in Equation (9), one obtains:

$$x_{\mathrm{w}}\left(\mathrm{d}\ln\gamma_{\mathrm{w}} + \frac{\mathrm{d}\,x_{\mathrm{w}}}{x_{\mathrm{w}}}\right) + x_{\mathrm{iso}}\left(\mathrm{d}\ln\gamma_i + \frac{\mathrm{d}x_i}{x_i}\right) = 0 \tag{S2}$$

Differentiating Equation (7) yields:

$$\mathrm{d}\ln\gamma_{\mathrm{w}} = 2A(1 - x_{\mathrm{w}})(-\mathrm{d}\,x_{\mathrm{w}}) \tag{S3}$$

By recalling that $x_j = K_{\mathrm{x},ji}x_i$:

$$1 - x_{\mathrm{w}} = \sum_{j=1}^{N} x_j = \sum_{j=1}^{N} K_{\mathrm{x},ji}x_i = x_i \sum_{j=1}^{N} K_{\mathrm{x},ji} \tag{S4}$$

x_i can therefore be expressed as (special case: $K_{\mathrm{x},ii} = 1$):

$$x_i = \frac{1 - x_\mathrm{w}}{\sum_{j=1}^{N} K_{\mathrm{x},ji}} = \frac{1 - x_\mathrm{w}}{1 + \sum_{\substack{j=1 \\ j\neq i}}^{N} K_{\mathrm{x},ji}} \tag{S5}$$

Differentiating Equation (S5) and dividing by x_i yields:

$$\frac{\mathrm{d}\,x_i}{x_i} = \frac{-\mathrm{d}x_\mathrm{w} \sum_{j=1}^{N} K_{\mathrm{x},ji} - (1 - x_\mathrm{w}) \sum_{j=1}^{N} \mathrm{d}\,K_{\mathrm{x},ji}}{(1 - x_\mathrm{w}) \sum_{j=1}^{N} K_{\mathrm{x},ji}} \tag{S6}$$

Plugging Equation (S6) in Equation (S2) yields, after little rearrangements, Equation (10). A summary of the main assumptions for the derivation is reported below:

- the system consists of N isomers in chemical equilibrium and one solvent, that without loss of generality is considered to be water.
- constant temperature and pressure.
- the water activity coefficient is well described by the 2-suffix Margules expression (also known in the literature as Porter equation or Norrish equation).

The integration of Equation (10) for $N > 2$ is not trivial. if, for instance, $N = 3$ and $i = 1$, Equation (10) is rewritten as:

$$\mathrm{d}\ln\gamma_1 = 2Ax_\mathrm{w}\mathrm{d}x_\mathrm{w} + \frac{\mathrm{d}K_{\mathrm{x},21}}{1 + K_{\mathrm{x},21} + K_{\mathrm{x},31}} + \frac{\mathrm{d}K_{\mathrm{x},31}}{1 + K_{\mathrm{x},21} + K_{\mathrm{x},31}} \tag{S7}$$

The integration of the second and third terms requires the knowledge of the functional relationship between $K_{\mathrm{x},31}$ and $K_{\mathrm{x},21}$. This relationship exists because, according to the phase rule (Equation (2)), both $K_{\mathrm{x},31}$ and $K_{\mathrm{x},21}$ are functions of x_w. However, the exact nature of this dependence varies for each specific system under investigation.

2 Dissolution modelling: approaching solid-liquid equilibrium

In this section the dissolution in the first time instants, when the system is away from solid-liquid equilibrium conditions, is discussed. At each time step, Equation (23) is still used to estimate $x_{\alpha,\mathrm{sat}}$

using the current bulk x_w, under the modelling assumption that the thin layer of liquid surrounding the crystal is always at solid-liquid equilibrium.

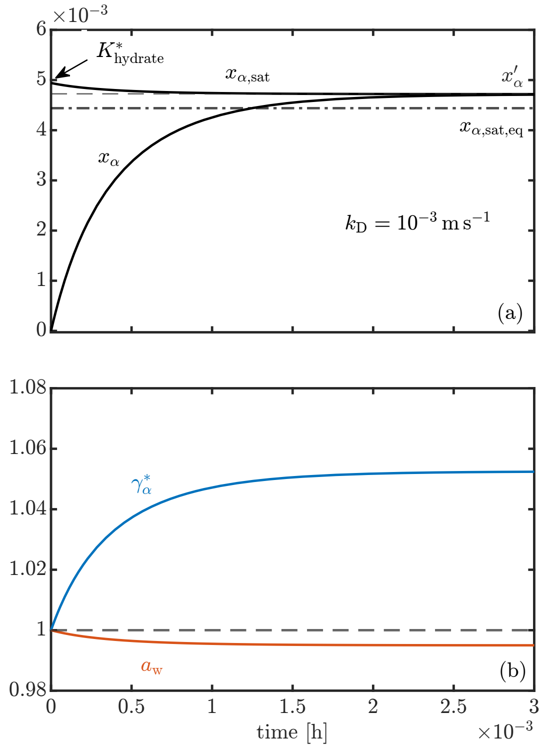

Figure S1: (a) zoom of Figure 4 (c) (here on a molar fraction basis) of the dynamics of α-lactose molar fraction, x_α, as predicted by the model during the initial transient approaching solid-liquid equilibrium conditions. (b) zoom of Figure 4 (e).

Figure S1 shows the evolution of x_α (a) and of γ_α^* and a_w (b) in the initial transient when solid-liquid equilibrium is not yet established. Before the dissolution starts, the system consists of pure water and Equation (21) states that $x_{\alpha,\mathrm{sat}}$ is equal to K^*_hydrate. However, x_α never reaches K^*_hydrate, because upon dissolution γ_α^* increases and, therefore, $x_{\alpha,\mathrm{sat}}$ decreases to satisfy Equation (21). The system reaches solid-liquid equilibrium at an intermediate value $x'_\alpha < K^*_\mathrm{hydrate}$ such that the dissolution driving force in Equation (20) approaches zero, as outlined below:

$$x_\alpha = x'_\alpha < K^*_\mathrm{hydrate} \quad \text{s.t.} \quad x_{\alpha,\mathrm{sat}} - x'_\alpha \approx 0 \tag{S8}$$

Dissolution takes place in the order of seconds, in comparison to mutarotation that requires 8 to 12 hours to reach equilibrium. The amount of β-lactose in the first seconds of the simulation is negligible and the system consists only of α-lactose and water such that $x_\alpha = x'_\alpha$ and $x_\mathrm{w} \approx 1 - x'_\alpha$. Introducing Equation (23) in Equation (S8) yields one equation in one unknown, x'_α, that corresponds to the highest molar fraction of α-lactose that satisfies solid-liquid equilibrium, as shown below:

$$x'_\alpha \, \gamma^*_\alpha\big|_{1-x'_\alpha} a_\mathrm{w}\big|_{1-x'_\alpha} - K^*_\mathrm{hydrate} = 0 \tag{S9}$$

The aforementioned feature could not be observed with our experimental apparatus due to the smaller time scale of dissolution with respect to the time needed for a chromatographic measurement (around 5 minutes). In the dissolution model developed in Section 4, the dissolution kinetic constant, k_D, and the initial crystal population, n_seed, have negligible impact on model predictions (Figure 4) if and only if they are chosen to guarantee that solid-liquid equilibrium is established significantly faster than mutarotation. The decreasing trend shown by $c_{\alpha,\mathrm{sat}}$ (mass fraction basis) in Figure 4 (c) spanning 8 to 12 hours refers to the slow mutarotation building up the β-lactose concentration that progressively makes c_α decrease from c'_α to $c_{\alpha,\mathrm{sat,eq}}$.

3 Estimation of F according to Visser

Table S1: Estimation of F by Visser[10] using the data from Talley and Hunter[24] and Nickerson and Moore[25].

Source	$\mathrm{g\,g_w^{-1}}$	$\mathrm{g\,g_{tot}^{-1}}$	$\mathrm{mol\,mol_{tot}^{-1}}$
Nickerson and Moore[25]	0.0346	0.1596	0.0434
Talley and Hunter[24]	0.0348	0.1118	0.0222

4 Experimental data on the effect of foreign sugars on lactose solubility

The solubility of α-lactose monohydrate as a function of dissolved sucrose at 25 °C is reported by Nickerson and Moore[25]. Table 2 of their manuscript reports the solubility of lactose as a function

of sucrose content relative to the lactose solubility in distilled water at the same temperature. We tested experimentally and confirm the validity of the solubility correlation for α-lactose monohydrate in water reported by Butler[29]:

$$c_{\mathrm{tot,sat,eq}} \left[\mathrm{g\,g_w^{-1}}\right] = \exp\,(2.389 + 0.028\,T\ [^{\circ}\mathrm{C}]) \tag{S10}$$

The Solubility of α-lactose monohydrate as a function of dissolved glucose and galactose is reported by Talley and Hunter[24]. To make the expression consistent with Equation (S10) for the limiting case of lactose-water solutions, a systematic correction of 0.005 is added:

$$c_{\mathrm{tot,sat,eq}} \left[\mathrm{g\,g_{tot}^{-1}}\right] = 0.18 - 0.2452\,c_{\mathrm{glucose}} \left[\mathrm{g\,g_{tot}^{-1}}\right] - 0.2477\,c_{\mathrm{galactose}} \left[\mathrm{g\,g_{tot}^{-1}}\right] \tag{S11}$$
